# Medical and Philosophical Intelligence

**Published:** 1819-12

**Authors:** 


### Medical and Philosophical Intelligence.

PROCEEDINGS *of the Royal Society of Edinburgh, Feb.* 15, 1819.— THE continuation of a paper, by Dr. WILLIAM ALISON, was read, entitled "Observations on some late Inquiries into the Physiology of the Nervous System; particularly into its Connexion with Muscular Motion, Secretion, and Animal Heat."

"The leading doctrine of this paper is, that there is no proof in the writings of physiologists, of the nervous system being essentially concerned in any of those functions of the living body which are performed without the intervention or consciousness of the mind,—that is, in any of the functions which constitute the organic life of Bichat and others; that the direct dependance of any of these functions, or any agency of the nervous system, is, in the present state of our knowledge, an hypothesis, unsupported by direct facts, and contradicted by much probable evidence; and that the office of the nervous system, in the natural and healthy state, may be said to be merely to minister to the wants of the mind, and maintain the connexion between mind and body.

The author arrives at this conclusion from an examination of the different classes of facts which have been at different times ascertained by physiologists, in regard to the connexion of changes in the nervous system, with muscular motion, secretion, and animal heat. In regard to the first of these, it appears clearly that two kinds of effects upon muscles have been observed to result from changes in the nervous system:

1. That the contractions of many muscles may be directly excited by such changes; and 2, that the irritability, or tendency to contraction in certain given circumstances, of all muscles, may be variously modified by such changes. The first kind of agency appears to be exerted solely on the voluntary, the last chiefly on the involuntary, muscles. But it likewise appears, from all the facts hitherto ascertained on the subject, that the irritability of muscular fibres on the living body is inherent with themselves, and is in no case directly dependent on any agency of nerves. So far there is no material difference between the doctrine here maintained and that of HALLER and his followers, which has been lately espoused, and strongly supported, by Dr. Wilson Philip.

The observations made by this last gentleman on the different effects of physical agents, applied to large and to small portions of the brain, on the two great classes of muscular organs; the former affecting chiefly the actions of the involuntary, and the latter particularly when touching the origins of the nerves exciting the voluntary muscles; have induced the author of this paper to add one to the many conjectures already hazarded in regard to the use of the ganglia and of the great sympathetic nerve, from which the involuntary nerves are chiefly, and many of the voluntary are partly, supplied. These he supposes to concentrate and to increase the influence, upon the organs which they supply, of changes which extend to large portions of the nervous system, by those sensations, emotions, and passions of mind, which are known particularly to affect these muscles, both voluntary and involuntary, which are supplied with nerves from the ganglia; and thence concludes, that the transmission of the effect of such things may be the principal object of this arrangement of nerves.

In regard to the connexion of the nervous system with secretion, the author contends, in opposition to several eminent physiologists, 1, that the nature of the actions of nerves is probably not galvanic; and, 2, even if they be galvanic, that these actions appear, from facts already known, not to be essentially concerned in secretion. And, in regard to its connexion with animal heat, he contends, 1, that no necessary connexion between the evolution of animal heat and any influence that can be derived from the nerves, has been established by the recent enquiries on this subject; and, 2, that the other cause, commonly assigned, since the time of Black and Crawford, for this function of animals, certainly exists, and is adequate to the explanation of the phenomena.

If it can be ascertained, by this kind of examination of the facts ascertained in this department of physiology, what functions are merely subject to the occasional control, and what are permanently dependant on the condition of the nervous system, the author conceives that two advantages to the science will result: 1, That greater simplicity and precision will be given to our views of the office of the nervous system; and, 2, that a more systematic arrangement of the science than has been usually attempted, will be authorized.

The animal life of Bichat,—sensation, thought, voluntary and instinctive motion, are manifestly dependant on the condition of the nervous system, and therefore on the functions of organic life, by which that condition is maintained. What may be called the chemical functions of organic life,—secretion, nutrition, &c. are manifestly dependant on the vital property of irritability, is residing in the muscles of involuntary motion; but this property of irritability has only an indirect dependance, either on the rest of the organic functions, or on any of the animal; on the former for the supply of blood, and on the latter for the arterialization of the blood supplied. There may be irritability where no action of nerves, and even where no nutrition, is going on; but there can be no nutrition, and no action of nerves, without irritability. Hence the exercise of this property appears to be the most fundamental of all the functions of living bodies; and it seems as reasonable and expedient to begin the study of the animal economy by an examination of its laws, as to begin the study of the actions of a machine at the point where the moving power is applied."

We take the foregoing abstract from the second Number of the Edinburgh Philosophical Journal, and give it here, because it is, in a manner, a duty in us to let our readers know that such opinions have been published. We cannot, of course, offer any judgment on the value that may be attributed to them, because we do not know on what facts the arguments for this doctrine may have been founded; but they must be new and very extraordinary ones that can render such a paradox feasible, in opposition to what is known on this subject by those who have thoroughly investigated it.

---

We were obliged to close the department for Critical Analyses in the present Number without having noticed an elegantly-written and erudite work, by Dr. Adam Hunter, on some mineral springs that have recently been discovered at Harrowgate, and on those of Thorp-Arch and Ilkley,* that has for some months past been urgently requiring our attention: we shall, then, in this place, adduce a few detached extracts from it, to show the properties of the waters, and recommend this interesting work to the perusal of our readers; for it is not susceptible of an analysis in a concise form, the detail of the chemical experiments necessarily occupying considerable space; and, on treating of the qualities of the waters, the author has so connected his remarks on those in particular, with reflections on the use of saline waters, bathing, &c. in general, and the natural history of several others of similar qualities, illustrated by the observations of many of the best writers on the subject, that we rather desire, indeed, that our readers should participate the pleasure we have enjoyed in its perusal, than to be the means of depriving them of it, by furnishing them with what they might think an analysis of the contents of this valuable essay.

"The two springs, the analysis of which is now presented to the public, were discovered about seven months ago, by boring in search of sulphur-water to supply the increasing demand for the baths. They are situated at the distance of about 200 yards from the promenade-room, in a field adjoining the Leeds and Ripon road, the property of Mr. Oddy, of the Lodge, at Low Harrogate. They have a north-east aspect, and command a pleasant view of the Lodge and neighbouring valley, which is tastefully laid out, and ornamented with clumps of trees.

"*New Saline Chalybeate Spring at Harrowgate.—Natural Properties.* The water newly taken from the spring is transparent, and has rather a sparkling appearance when poured from one glass into another; the taste is distinctly saline, but not unpleasantly so, compared with the sulphur-water. The temperature of the well was 47° Fahrenheit, the thermometer in the shade standing at 62°; and, being immersed in a pool of water in the immediate neighbourhood, it stood at 58°. Its specific gravity is 1·0075.

—"Upon summing up the various products in the foregoing analysis, the wine-gallon will be found to contain: Muriate of soda, 434,00 grains; muriate of lime, 30·00; muriate of magnesia, 13·00; sulphate of lime, 9·00; carbonate of iron, 5·00; carbonate of lime, 3·00; loss, 2·50:—total, 496·50.

"*Gaseous Contents.*—Carbonic acid gas, 6·320 cubic inches; azotic gas, 3·970; oxygen gas, 0·870:—11·160.

"*New Chalybeate Spring.—Sensible Properties.* The appearance of the water, when first taken from the well, is remarkably clear and bright; no perceptible smell can be detected; it sparkles gently when poured from one glass into another; when at rest, air-bubbles slowly separate, and adhere to the sides of the glass; the taste is distinctly and strongly chalybeate. The temperature was 48° Fahrenheit, the surrounding atmosphere varying from 58° to 64°. Specific gravity, 1·0012.

—"From these experiments it is evident, that iron is the predominant ingredient in this water. The proportions in a wine-gallon being, of

* *An Essay on two Mineral Springs, recently discovered at Harrowgate; and on the Springs of Thorp-Arch and Ilkley: including the History, Chemical Analysis, and Medicinal Properties of these Waters; with some Observations on their Use.* By Adam Hunter, M.D. F.R.M.S.E. Physician to the House of Recovery at Leeds. 8vo. pp. 134. Robinson and Co. Leeds; Hurst, Robinson, and Co. London. 1819.

carbonate of iron, 10·50 grains; muriate of soda, 2·50; muriate of magnesia, a very minute quantity. Carbonic acid gas, 16·500 cubic inches; azotic gas, 4·200:—total, 20·700.

"*On the Mineral Spring at Thorp-Arch.*—Thorp-Arch is beautifully situated upon the banks of the River Wharfe, between Tadcaster and Wetherby, and at nearly an equal distance of 12-13 miles from Leeds, Harrogate, and York.

"*Chemical Analysis.*—When recently drawn from the pump, the water has a clear sparkling appearance, but on standing a short time becomes slightly turbid. It has a distinct saline taste, not unsimilar to the saline chalybeate spring at Harrogate, which it also resembles in its other sensible properties. Temperature 49°, the surrounding medium being 57°. Specific gravity, at 55°, 1·0095.

"The wine-gallon contains, muriate of soda, 562·00 grains; muriate of lime, 12·25; muriate of magnesia, 7·25; carbonate of iron, 1·75; silica, 0·75;—584·00. *Gaseous contents:* Carbonic acid, 10·56 cubic inches; azotic gas, 6·00:—16·56.

"The component parts of this spring do not differ materially from those of the sulphur and saline chalybeate waters at Harrogate. It contains a smaller proportion of saline matter than the former, and less iron than the latter; while, at the same time, it partly combines the properties of both. Though the sulphuretted hydrogen gas, with which it was formerly said to be impregnated, has now disappeared, it not only retains the same quantity, but, by the present analysis, seems to have acquired an additional proportion of saline ingredients.

"*Observations on the Ilkley Fountain.*—The village of Ilkley lies on the south side of the Wharfe, upon the road between York and Lancaster, about sixteen miles west from Thorp-Arch, six miles from Otley, and sixteen from Leeds; while at a considerable distance to the north-west lies the ancient town of Skipton.

"The wine-gallon was found to contain, muriate of lime, 6·50 grains; muriate of magnesia, 3·00;—total, 9·50. *Gaseous contents:* Carbonic acid gas, 12·60 cubic inches; atmospheric air, 5·40:—18·00."

---

We take the following account from the *Journal der Practischen Heilkunde des Berlin,* for May 1819: it will be generally perused with pleasure, we think, for various reasons.

*The Celebration of* Jenner's Festival *at Berlin, in the Year* 1819.—On the 14th of May, the physicians of Berlin, according to custom, celebrated in the most joyful manner that day, appropriated to the remembrance of the practice of cow-pox inoculation, and of its illustrious discoverer, Edward Jenner. The report, made by the Institution, of the progress of vaccination in the Prussian monarchy, during the year 1817, was of the most gratifying kind. The whole number inoculated was 307,596; of which number there were within the extent of the administration of Berlin, 5,353 (2,600 of these in the Royal Vaccine Institution); at Königsberg 22,685, Grunbinnen 24,525, Danzig 12,458, Marienwerder 18,169, Potsdam 16,044, Frankfurth 17,900, Stettin 13,826, Coslin 1,016, Stralsund 4,829, Breslau 17,816, Liegnitz 16,124, Reichenbach 14,658. Oppeln 17,448, Posen 26,086, Bromberg 18,144, Magdeburg, 9,007, Merseburg 13,269, Erfurt 7,364, Münster 4,550, Minden 10,737, Cleve 4,498, and Coblenz 10,990. The accounts from

Arensberg, Colln, Düsseldorf, Aschen, and Trier, were not less agreeable.

*Prize Question.*—The Royal Academy of Sciences of Paris proposes as a prize question, to be decided in 1821, the following:

"To give a comparative description of the brain in the four classes of vertebral animals, and particularly in reptiles and fishes, endeavouring thence to recognize the analogy of the different parts of this organ; marking with care the changes of form and of proportion they present, and following, as profoundly as possible, the roots of the cerebral nerves.

"It will be sufficient to make the observations on a certain number of genera, chosen in the principal natural families of each class; but it will be necessary that the principal preparations be represented by drawings sufficiently detailed, that they may thence be reproduced, and their accuracy determined."

The premium will be a golden medal of the value of three thousand francs; it will be appropriated in the public sitting of the month of May 1821. The utmost term for the transmission of memoirs, legibly written in French or in Latin, is the 1st of January, 1821. They should be addressed, free of carriage, to the Secretary of the Institute.

### REPORT OF DISEASES.

THE remarkable severity of the present season has produced the ordinary effects of such a temperature of the atmosphere in great cities, which are generally more severe and frequent here than in the open country. In the latter the temperature is nearly similar in different localities; but in cities, especially those, like London, not built in regularly extended streets, the active walker will in some parts glow with warmth, whilst a sudden turn will expose him to a current that will chill him by its cold. This will in some degree, perhaps, explain why persons of robust habits, who bear the climate of our island generally without inconvenience, are so commonly affected with pulmonary disorders and rheumatism when in London. These, with hooping-cough in children, which is particularly prevalent in the south-western district, are the reigning maladies. Laryngitis has been frequent, too, in adults; though the inflammation, in most cases, extends throughout the whole bronchiæ. We cannot too forcibly inculcate the use of tartar-emetic, after blood-letting, &c. and with blisters, in the latter affection: but, we repeat, it is only in very large and frequent doses that its good effects will be fully experienced. Many medical practitioners, with whom we have discoursed on the use of this remedy, hear with scepticism that it may be administered in doses of half a grain, and frequently a grain, to adults, repeated every two or three hours, without causing vomiting, after one or two attacks of that action, especially if the bowels have previously been purged; but experience has convinced several of them of the truth of this statement, and disclosed to them a remedy of very considerable value, which they had previously thought but lightly of, from its having been exhibited in insufficient quantity. This must be remarked: the use of it in this way must be accompanied with assiduous attention to its effects, as very great debility not unfrequently comes on with great rapidity; but that produced by excessive blood-letting in intense inflammatory affections, is often equally great, more permanent, and very often much less efficacious.

As no other particular remarks occur to us on the prevalent diseases, we shall terminate this Report with some further assertions respecting tartar-emetic. In diseases of the heart accompanied with excitement, joined with digitalis, and sometimes with belladonna, &c.; and in many forms of disease of the liver of an inflammatory character, with calomel, sulphate of magnesia occasionally, and the other ordinary measures; the efficacy of tartar-emetic merits the warmest praise. Here, of course, it must be given in much smaller doses.

### METEOROLOGICAL JOURNAL.

By Messrs. William Harris and Co. 50, Holborn, London.

*From October 20 to November 19, inclusive.*

| Day of Month. | Moon. | Rain guage | THERM. | | | BAROM. | | De Luc's HYGROM. Dry | Damp | | WIND. | | ATMOSPHERIC VARIATION. | | |
|---|---|---|---|---|---|---|---|---|---|---|---|---|---|---|---|
| Oct. | | | | | | | | | | | | | | | |
| 20 | | ,35 | 53 | 55 | 42 | 29·72 | 29·60 | | 16 | 19 | SW | NW | Cloud. | Rain | Rain |
| 21 | | ,14 | 44 | 38 | 30 | 29·54 | 29·38 | | 14 | 15 | NW | SE | Cloud. | Snow | Rain |
| 22 | | | 33 | 40 | 38 | 29·46 | 29·50 | | 11 | 14 | NW | W | Snow | Rain | Cloud. |
| 23 | | | 41 | 45 | 38 | 29·40 | 29·40 | | 15 | 14 | W | N | Fine | Cloud. | Cloud. |
| 24 | | | 41 | 47 | 37 | 29·40 | 29·45 | | 13 | 12 | N | N | Fine | | |
| 25 | | | 40 | 44 | 35 | 29·55 | 29·58 | | 11 | 11 | NW | W | Fine | | |
| 26 | ☾ | | 46 | 50 | 34 | 29·66 | 29·82 | | 12 | 10 | N | NW | Rain | Fine | Fine |
| 27 | | ,06 | 39 | 44 | 33 | 29·85 | 29·84 | | 11 | 13 | NNW | NE | Fine | Rain | Cloud. |
| 28 | | | 36 | 40 | 35 | 29·81 | 29·78 | | 12 | [illegible] | NE | NE | Fine | | |
| 29 | | ,80 | 37 | 40 | 39 | 29·57 | 29·56 | | 12 | 13 | NE | ENE | Cloud. | Rain | Rain |
| 30 | | ,66 | 41 | 42 | 46 | 29·57 | 29·70 | | 14 | 16 | NE | NE | Rain | | |
| 31 | | ,50 | 49 | 50 | 46 | 29·86 | 29·92 | | 20 | 16 | E | NE | Rain | Rain | Fine |
| Nov | | | | | | | | | | | | | | | |
| 1 | | ,02 | 47 | 47 | 44 | 29·86 | 29·76 | | 17 | 15 | E | NNE | Rain | Cloud. | Fine |
| 2 | ○ | | 46 | 49 | 39 | 29·78 | 29·77 | | 13 | 12 | N | NW | Fine | | |
| 3 | | | 44 | 46 | 40 | 29·97 | 30·05 | | 11 | 12 | NW | W | Fine | | |
| 4 | | | 46 | 49 | 48 | 29·96 | 29·90 | | 12 | 14 | W | W | Fine | | |
| 5 | | ,17 | 51 | 54 | 47 | 29·73 | 29·57 | | 13 | 16 | SW | W | Cloud. | Cloud. | Rain |
| 6 | | | 51 | 55 | 44 | 29·52 | 29·44 | | 15 | 12 | W | WSW | Fine | | |
| 7 | | | 46 | 47 | 39 | 29·43 | 29·56 | | 12 | 11 | W | W | Fine | Cloud. | Cloud. |
| 8 | | | 42 | 44 | 34 | 29·63 | 29·80 | | 11 | 10 | NE | N | Fine | | |
| 9 | ☽ | | 37 | 40 | 44 | 29·91 | 29·64 | | 11 | 11 | WNW | WSW | Fine | | |
| 10 | | ,15 | 48 | 50 | 44 | 29·40 | 29·44 | | 12 | 15 | WSW | NNE | Fine | Fine | Rain |
| 11 | | ,24 | 46 | 47 | 39 | 29·73 | 29·95 | | 15 | 13 | NE | ENE | Cloud. | Rain | Rain |
| 12 | | ,05 | 44 | 47 | 43 | 29·97 | 29·90 | | 12 | 11 | ENE | NE | Fine | Fine | Rain |
| 13 | | | 46 | 49 | 44 | 29·83 | 29·80 | | 12 | 12 | E | NNE | Cloud. | | |
| 14 | | | 46 | 48 | 44 | 29·83 | 29·83 | | 14 | 13 | N | NW | Fine | Fine | Cloud. |
| 15 | | ,10 | 46 | 48 | 42 | 29·76 | 29·60 | | 14 | 14 | WNW | N | Cloud. | Cloud. | Rain |
| 16 | | ,49 | 43 | 44 | 39 | 29·55 | 29·55 | | 15 | 14 | NNE | NE | Rain | | |
| 17 | ● | ,09 | 40 | 41 | 42 | 29·60 | 30·00 | | 13 | 15 | E | ENE | Cloud. | Rain | Cloud. |
| 18 | | | 44 | 46 | 34 | 30·10 | 30·10 | | 11 | 9 | E | NE | Fine | | |
| 19 | | | 37 | 39 | 36 | 29·98 | 29·85 | | 10 | 9 | NE | N | Cloud. | | |

The quantity of rain fallen in October, is 2 inches and 51-100ths.

*** *In order that the next half-yearly History of the Progress of Medicine may comprise a complete account of the medical literature of England to the close of the present year, and that of Foreign Nations as nearly as possible to the same period, without interfering with the usual Monthly Number of the Journal, to the exclusion of other valuable matter, it is intended to publish a Supplementary or extra Number in January. The* Title *and* Index *will in future be given in the Supplements.*

*To prevent disappointment, the Subscribers will please to give their Booksellers directions to order their Correspondents to forward the Supplementary Number with the current one.*

☞ *The Publisher informs the Subscribers to this Journal, that the* General Index, *or Analytical Table, of the first* Forty Volumes, *will certainly be ready for delivery on the 15th of December; and, as very few more Copies than what are subscribed for have been printed, it is necessary for those who wish to obtain a Copy to order it immediately, through the Booksellers or otherwise.*

*Want of Room obliges us to defer the* List of Books *until the next Number.*

